# USING THE COLLECTIVE IMPACT FRAMEWORK TO REFRAME AGING IN MISSISSIPPI

**DOI:** 10.1093/geroni/igac059.3125

**Published:** 2022-12-20

**Authors:** Kina White, Kaye Bender, David Buys

**Affiliations:** Mississippi State Department of Health, Ridgeland, Mississippi, United States; Mississippi Public Health Association, Jackson, Mississippi, United States; Mississippi State University, Ms State, Mississippi, United States

## Abstract

Trust for America’s Health (TFAH), the Mississippi State Department of Health (MSDH), and the Mississippi Public Health Association (MPHA) partnered to organize the Mississippi Age-Friendly Public Health System (AFPHS) Work Plan. Built on the foundation of Reframing Aging training offered by the Gerontological Society of America in May 2021, the Work Plan aims to reframe how Mississippi’s public health system addresses aging using the AFPHS 6Cs. Developed by the MS AFPHS Advisory Committee using a collective impact framework, the Work Plan has now been opened for public comment prior to being finalized for adoption across partner agencies. An AFPHS focuses on promoting health; preventing injury; managing chronic conditions; optimizing physical, cognitive, and mental health; and facilitating social engagement for older adults. The Work Plan’s purpose is to guide MSDH and partner agencies in implementing an AFPHS for Mississippi. This is Phase One of a comprehensive age-friendly movement for Mississippi, with the next phase being an age-friendly state. Much of the work that MSDH will accomplish under this plan lays the foundation for the state to then have many age and dementia friendly communities. This presentation will discuss how Mississippi is reframing aging through collective impact and AFPHS 6Cs frameworks; the results of the public vetting of the Work Plan; and the final Work Plan components based on the public vetting. The process and its results can be replicated in other rural states where resources for aging populations are limited and historical perspectives on healthy aging are open to change.

